# Discourse on the Enlarged and Pendulous Abdomen, &c. Augmented by a Dissertation on Gout, Suggesting New Physiological Views of Its Cause, &c.

**Published:** 1842-07

**Authors:** 


Art. III.—*Discourse on the Enlarged and Pendulous Abdomen, &c. Augmented by a Dissertation on Gout, suggesting New Physiological Views of its Cause, &c.* By Richard Frankum, Esq. Surgeon.—*London,* 1842. 12mo, pp. 121.

A work of idler and shallower gossip, of triter commonplaces, and containing more slight and more ridiculous pathology, than that of Mr. Frankum, we have seldom set our eyes on. The singular originality of some parts has struck us. Thus (pp. 27-8), "The digestive power of the stomach is, in general, proportionate to the quantity of food given to it. . . . The natural powers, then, of the stomach, are in proportion to the quantity of food it has to feed on, as the size of a blacksmith's arm increases according to the strength demanded from it." An agreeable piece of information this, for those unruly patients, who, with weak stomachs, have unmanageable appetites, and who have only to become gourmands in order to rid themselves of their atonic dyspepsia.

The humanity and tenderness as well as the ingenuity with which Mr. Frankum argues the cause of the unfortunate owners of pendulous bellies, are creditable alike to his head and heart.

"Before," he says, "we proceed to the treatment of this disorder, we shall say one word of the attitude of those who have a protuberant or pendulous belly, in justice to those, and many are they, whose feelings are anything but ostentatious, as their gait implies. It is certainly somewhat odd that presumed dignity of personal appearance should be derived from unnatural causes, and that the erect position of the body is conceived to be the *most dignified and imposing when corporeal substance and a projecting form are given to it.* All this is not less curious than true; and the strut of a man thus circumstanced is attributed to any but the right cause, viz., the infirmity of his condition. None but those who feel and suffer from this infirmity can understand the pain which modest minds are subject to endure from the construction which the world is too apt to put upon it. . . . Under all circumstances, it is a case that requires the kindliest instead of the harshest construction, and the silence of commiseration rather than the finger of remark." (pp. 41, 43.)

What Gibbon calls the "chivalry" of Burke's defence of the church and the clergy in his reflections on the French revolution, was nothing to this!

"Gout," Mr. Frankum tells us, p. 121, "is a disease essentially dependent on an inflammatory affection of the mucous membrane of the stomach, and its degrees of severity are also dependent upon the extent to which that organ is affected." This, we presume, is the "new physiological (?) view" announced in the title-page. But from other parts of Mr. Frankum's volume, we should scarcely have expected that he should have so specially and formally set himself forth as a supporter of the theory of an intimate relation between stomach disease and gout: for what do we find him saying at p. 27?—"There is no mistake more common than that a large eater has always a full and foul stomach; the interference with health seldom arises from an affection of this most vital of our organs, supposing it to be in a healthful condition!" And, in the second page following, he speaks "of the insusceptibility of the stomach to take on diseased action." We are informed at p. 114 that "purging is one of the best and safest diaphoretics" in gout.

In the foot-note references to this volume non-professional authors are more frequently quoted than professional. Schoolboy citations from Shakspeare occur repeatedly. Byron's Narrative, Childe Harold, Boswell's Life of Johnson, Bruce's Travels, Bacon's Essays, Middleton's Life of Cicero, are dragged on the arena, on occasions when assuredly the *nodus non vindicibus talibus dignus est.* We regret the appearance of such a work, and trust that our present notice of it will make apparent to Mr. Frankum the error he has committed in giving a production so totally unprofessional and so trashy to the public.

